# Association of age at menopause with cardiovascular disease and mortality: insights from NHANES and a clinical cohort

**DOI:** 10.3389/fcvm.2026.1797019

**Published:** 2026-06-23

**Authors:** Xinmiao Hong, Linxi Jin, Guoli Shang, Qiwei Chen, Zhuang Han, Shuhong Yao, Zhita Wang, Liang Li, Weidong He, Xianpei Heng, Liuqing Yang

**Affiliations:** 1 Department of Endocrinology, People's Hospital Affiliated to Fujian University of Traditional Chinese Medicine, Fuzhou, Fujian, China; 2 First Clinical Medical College, Fujian University of Traditional Chinese Medicine, Fuzhou, Fujian, China; 3 Department of Geriatrics, People's Hospital Affiliated to Fujian University of Traditional Chinese Medicine, Fuzhou, Fujian, China

**Keywords:** age at menopause, all-cause mortality, cardiovascular disease, cardiovascular mortality, mediation analysis

## Abstract

**Background:**

Age at menopause is closely associated with cardiovascular disease and mortality, whereas the underlying mechanisms remain unclear. This study employed mediation analysis to explore potential pathways underlying this association.

**Methods:**

Natural postmenopausal women were identified from the NHANES database to assess the association between age at menopause and cardiovascular disease and mortality. Mediation analysis was performed to identify potential mediators. A hospital-based cohort was used for a supportive analysis.

**Results:**

Compared with women with menopause at 50–51 years, those with menopause before 40 years had a higher prevalence of cardiovascular disease and increased risks of all-cause and cardiovascular mortality. Dose–response analysis indicated that each one-year decrease in age at menopause was associated with 3%, 3%, and 4% increases in cardiovascular disease prevalence, all-cause mortality, and cardiovascular mortality, respectively. Mediation analysis showed that total cholesterol (TC), triglycerides (TG), high-density lipoprotein cholesterol (HDL-C), hypertension, and glycated hemoglobin partially mediated the association between age at menopause and adverse outcomes. A hospital-based cohort study further supported these findings.

**Conclusion:**

Early menopause is significantly associated with higher cardiovascular disease prevalence, all-cause mortality, and cardiovascular mortality, and this association is partially mediated by metabolic dysregulation.

## 1 Introduction

Menopause is closely associated with multiple diseases, and early menopause may further increase the risk of these conditions (1, 2). Cardiovascular disease remains the leading cause of death among women (3). Elucidating the association between age at menopause and cardiovascular disease and mortality, as well as the underlying mechanisms, is of important clinical significance.

Women with early menopause often present with obesity, hypertension, and dysregulated lipid and glucose profiles, suggesting that metabolic dysregulation may be a key consequence of early menopause (4, 5). In addition, metabolic dysregulation plays a crucial role in the development and progression of cardiovascular disease (6). Hyperlipidemia promotes atherosclerosis and thrombosis, thereby increasing the risk of stroke and myocardial infarction (7). Hyperglycemia induces oxidative stress and inflammatory responses, resulting in endothelial injury and circulatory dysfunction and increasing the risk of cardiovascular disease and mortality (8, 9). However, the association between age at menopause and these adverse outcomes, along with the potential role of metabolic factors, remains incompletely understood. This study aimed to evaluate the association between age at menopause and cardiovascular disease and mortality, and to elucidate the underlying pathways.

In summary, this study was based on data from the National Health and Nutrition Examination Survey (NHANES) and focused on naturally postmenopausal women. The results showed that early menopause was significantly associated with higher cardiovascular disease prevalence, all-cause mortality, and cardiovascular mortality. Mediation analysis indicated that total cholesterol (TC), triglycerides (TG), high-density lipoprotein cholesterol (HDL-C), glycated hemoglobin, and hypertension partially mediated these associations. The hospital-based cohort showed consistent results with those of the NHANES analysis, further supporting the adverse impact of early menopause on cardiovascular health and strengthening the robustness of the findings. This study provides important evidence for the prevention and management of cardiovascular disease in postmenopausal women.

## 2 Materials and methods

### 2.1 Study population

**Figure 1:**
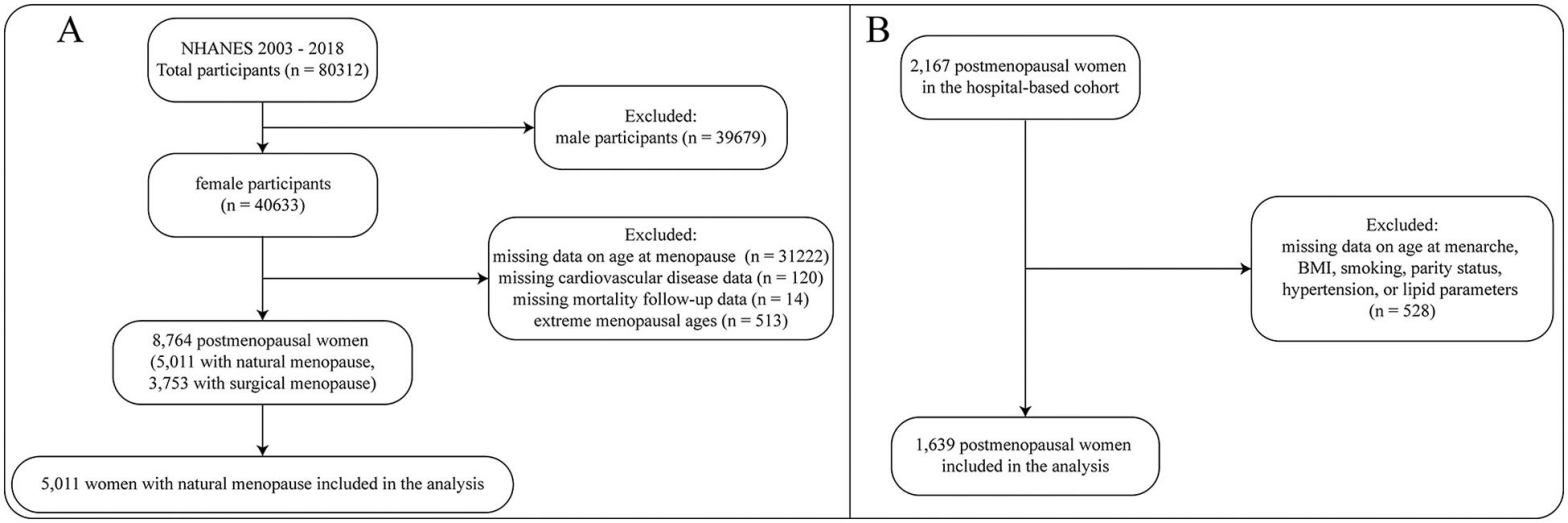
Flowchart of participant selection. NHANES cohort **(A)** Hospital-based cohort **(B)**.

The data for this study were derived from the NHANES database, whereas all-cause and cardiovascular mortality data were obtained from the National Death Index (NDI) database. A total of 80,312 participants were included from NHANES 2003–2018. After excluding 39,679 men and 31,869 participants with missing data on age at menopause, cardiovascular disease, or mortality outcomes, 8,764 postmenopausal women were finally included. Among them, 3,753 were surgical postmenopausal women (42.8%), and 5,011 were natural postmenopausal women (57.2%). This study focused on natural postmenopausal women for the final analysis (Figure 1A).

The hospital-based cohort data were obtained from the Research Integration Platform of the People's Hospital Affiliated to Fujian University of Traditional Chinese Medicine (10). A total of 2,167 naturally postmenopausal women were identified between 2022 and 2024. After excluding 528 participants with missing key clinical information, 1,639 patients were included in the supportive analysis (Figure 1B).

### 2.2 Exposure assessment

Naturally postmenopausal women were identified using the NHANES reproductive health questionnaire. Menopausal status was determined by asking participants whether they had menstruated within the past 12 months, with additional confirmation that amenorrhea was due to natural menopause. Age at menopause was defined as the self-reported age at last menstrual period.

Data from the National Institutes of Health indicate that the median age at menopause is 51 years. Accordingly, age at menopause was categorized for analysis as <40 years (premature menopause), 40–44 years (early menopause), 45–49 years (relatively early), 50–51 years (reference category), 52–54 years (relatively late), and ≥55 years (late menopause). This categorization enables effective discrimination of disease risk across different age-at-menopause groups (11).

### 2.3 Outcome ascertainment

The outcomes of this study included cardiovascular disease, all-cause mortality, and cardiovascular mortality. Cardiovascular disease was identified using the NHANES health status questionnaire. Participants were classified as having cardiovascular disease if they reported a physician diagnosis of coronary heart disease, congestive heart failure, angina, myocardial infarction, or stroke. Mortality outcomes were ascertained using the NDI database, with follow-up through 31 December 2019. All-cause mortality was defined as death from any cause, whereas cardiovascular mortality was defined using NDI underlying cause-of-death codes 54–68 and 70.

### 2.4 Covariates

To control for potential confounders, this study included demographic and lifestyle variables, such as age, age at menarche, race, educational level, body mass index (BMI), hormone therapy, parity status, and smoking.

Race was categorized as Mexican American, non-Hispanic White, non-Hispanic Black, and other; educational level was categorized as below high school, high school graduate, and above high school; BMI was categorized as <25 kg/m^2^, 25–29.9 kg/m^2^, 30–34.9 kg/m^2^, and ≥35 kg/m^2^.

### 2.5 Statistical analysis

#### 2.5.1 Main analyses

Statistical analyses were conducted using R software (version 4.5.1). Continuous variables are expressed as mean ± standard deviation, whereas categorical variables are presented as counts and weighted percentages. A two-sided *P* value < 0.05 was considered statistically significant.

All analyses accounted for sampling weights, stratification, and clustering according to the complex survey design of NHANES. Missing covariates were imputed using a random forest-based imputation method. Weighted logistic regression was used to assess the association between age at menopause and cardiovascular disease prevalence, whereas weighted Cox regression was used to evaluate associations with all-cause and cardiovascular mortality. Restricted cubic spline (RCS) models were further applied to evaluate dose–response relationships between age at menopause and the aforementioned outcomes. In addition, cardiovascular disease subtype analyses were performed, along with subgroup analyses for cardiovascular disease, all-cause mortality, and cardiovascular mortality.

#### 2.5.2 Mediation analysis

This study assessed the mediating effects of TC, TG, HDL-C, glycated hemoglobin, and hypertension in the association between age at menopause and cardiovascular disease and mortality, aiming to explore the potential role of metabolic factors.

#### 2.5.3 Sensitivity analyses

To enhance the robustness of the findings, four sensitivity analyses were conducted, including the inclusion of surgically postmenopausal women, the exclusion of participants who died within the first year of follow-up, the exclusion of participants with missing covariate data, and the use of alternative age-at-menopause categorization schemes.

### 2.6 Hospital-based supportive cohort

The hospital-based cohort was used as a supportive analysis to evaluate the generalizability of the NHANES findings. The study included naturally postmenopausal women. Cardiovascular disease was defined as coronary heart disease, heart failure, angina, myocardial infarction, or stroke. RCS models were used to evaluate the dose–response relationship between age at menopause and cardiovascular disease.

## 3 Results

### 3.1 Baseline characteristics of the NHANES population

A total of 5,011 naturally postmenopausal women were included in this study. Participants were categorized into six groups according to age at menopause: <40 years (*n* = 251), 40–44 years (*n* = 564), 45–49 years (*n* = 1,430), 50–51 years (*n* = 1,084), 52–54 years (*n* = 894), and ≥55 years (*n* = 788). Baseline characteristics are shown in Table 1.

**Table 1:** Baseline characteristics of women with natural menopause.

	Age at Menopause						
	<40	40–44	45–49	50–51	52–54	≥55	*P*
N	251	564	1,430	1,084	894	788	
Age (years)	63.3 (11.5)	63.9 (10.9)	62.2 (10.1)	64.8 (9.3)	64.6 (8.6)	67.9 (7.7)	<0.001
Age at Menarche (years)	12.8 (2.0)	13.0 (1.9)	12.9 (1.8)	12.9 (1.8)	12.9 (1.7)	13.1 (1.9)	0.302
Race, n (%)							<0.001
Mexican American	55 (8.9)	99 (6.4)	267 (7.0)	180 (5.2)	105 (3.5)	93 (3.0)	
Non-Hispanic White	91 (68.1)	246 (72.9)	584 (70.4)	489 (76.6)	422 (77.8)	405 (79.9)	
Non-Hispanic Black	56 (12.2)	105 (9.6)	282 (10.7)	174 (7.5)	163 (7.6)	149 (7.9)	
other	49 (10.7)	114 (11.1)	297 (11.9)	241 (10.7)	204 (11.1)	141 (9.2)	
Education, n (%)							<0.001
< High school	104 (25.5)	208 (22.4)	437 (17.5)	305 (14.7)	207 (11.9)	212 (14.9)	
High school	62 (32.1)	142 (26.7)	341 (24.2)	250 (25.9)	211 (24.2)	174 (23.0)	
> High school	84 (42.4)	214 (50.9)	650 (58.3)	527 (59.4)	474 (63.9)	401 (62.1)	
BMI, n (%)							0.643
<25.0	57 (28.5)	137 (30.1)	406 (30.7)	303 (31.2)	252 (27.7)	210 (33.2)	
25.0–29.9	72 (26.7)	192 (28.7)	421 (30.5)	323 (33.4)	273 (31.8)	235 (28.9)	
30.0–34.9	69 (21.1)	127 (20.2)	323 (19.7)	229 (20.0)	197 (22.2)	171 (21.5)	
≥35	48 (23.6)	101 (21.0)	255 (19.1)	218 (15.5)	155 (18.3)	163 (16.4)	
Hormone therapy, n (%)							0.003
No	194 (73.1)	441 (66.3)	1,091 (69.5)	837 (71.7)	675 (70.7)	536 (59.6)	
Yes	54 (26.9)	121 (33.7)	328 (30.5)	242 (28.3)	216 (29.3)	248 (40.4)	
Smoking, n (%)							<0.001
No	145 (53.0)	328 (51.0)	830 (51.7)	695 (62.9)	576 (63.5)	502 (61.7)	
Yes	106 (47.0)	236 (49.0)	600 (48.3)	388 (37.1)	317 (36.5)	285 (38.3)	
Parity status, n (%)							0.671
No	26 (13.9)	58 (10.2)	126 (11.3)	96 (9.6)	79 (10.3)	58 (8.7)	
Yes	225 (86.1)	506 (89.8)	1,304 (88.7)	988 (90.4)	810 (89.7)	730 (91.3)	
TC (mg/dL)	212.0 (45.3)	209.3 (43.6)	207.2 (41.2)	205.7 (40.4)	206.7 (41.6)	206.5 (40.8)	0.296
TG (mg/dL)	155.5 (101.0)	151.3 (96.5)	149.4 (94.4)	146.7 (92.7)	148.2 (93.5)	147.8 (91.3)	0.102
HDL (mg/dL)	56.9 (16.6)	57.6 (17.4)	59.3 (17.5)	61.7 (16.4)	60.3 (18.6)	60.6 (17.8)	0.043
HbA1c (%)	6.4 (1.4)	6.3 (1.2)	6.1 (1.1)	5.9 (1.0)	6.1 (0.9)	6.0 (1.1)	0.127
Hypertension, n (%)	166 (66.8)	351 (62.2)	830 (61.4)	637 (57.4)	549 (59.8)	467 (58.3)	0.015
Cardiovascular disease	55 (16.5)	85 (13.3)	164 (11.6)	159 (9.1)	93 (11.0)	114 (10.8)	0.006
All-cause mortality	59 (18.6)	121 (17.8)	211 (14.6)	183 (10.5)	139 (13.6)	140 (12.5)	0.003
Cardiovascular mortality	22 (8.4)	45 (7.5)	56 (6.4)	71 (4.8)	48 (5.7)	39 (5.1)	0.012

BMI, body mass index; TC, total cholesterol; TG, triglycerides; HDL, high-density lipoprotein cholesterol; HbA1c, glycated hemoglobin. Continuous variables are presented as mean ± standard deviation and categorical variables as number (weighted %).

Significant differences were observed in age, race, educational level, smoking, and HDL levels across the different age-at-menopause groups. A clear trend toward higher lipid levels and glycated hemoglobin, increased prevalence of hypertension and cardiovascular disease, and higher proportions of all-cause and cardiovascular mortality was observed across earlier age-at-menopause groups.

### 3.2 Associations of age at menopause with cardiovascular and mortality outcomes

#### 3.2.1 Categorical analyses

Model 2 served as the primary model adjusted for potential confounders, whereas Model 3 additionally incorporated metabolic factors as an extended model. Weighted logistic regression analysis showed that, compared with women who experienced menopause at 50–51 years, those with menopause before 40 years had a significantly higher prevalence of cardiovascular disease (OR = 1.57, 95% CI: 1.15–2.66). Weighted Cox regression analyses indicated that menopause before 40 years was associated with significantly higher risks of all-cause mortality (HR = 1.56, 95% CI: 1.08–2.39) and cardiovascular mortality (HR = 1.30, 95% CI: 1.07–2.34). These associations remained consistent after further adjustment for metabolic factors. Trend tests were statistically significant (Table 2).

**Table 2:** Cardiovascular disease prevalence and mortality by age at menopause.

Age at menopause	Number	Model1	Model2	Model3
Cardiovascular disease prevalence		OR (95% CI)	OR (95% CI)	OR (95% CI)
<40	55	1.89 (1.23, 3.15)	1.57 (1.15, 2.66)	1.54 (1.03, 2.60)
40–44	85	1.35 (0.95, 1.92)	1.23 (0.84, 1.79)	1.22 (0.85, 1.75)
45–49	164	0.90 (0.64, 1.28)	0.85 (0.59, 1.22)	0.82 (0.57, 1.19)
50–51	159	1	1	1
52–54	93	0.73 (0.50, 1.08)	0.76 (0.52, 1.12)	0.77 (0.52, 1.15)
≥55	114	0.88 (0.60, 1.27)	0.88 (0.60, 1.28)	0.85 (0.58, 1.25)
*P* for trend		<0.01	<0.05	<0.05
All-cause mortality		HR (95% CI)	HR (95% CI)	HR (95% CI)
<40	59	1.65 (1.16, 2.57)	1.56 (1.08, 2.39)	1.49 (1.02, 2.28)
40–44	121	1.29 (1.00, 1.67)	1.23 (0.94, 1.60)	1.22 (0.94, 1.59)
45–49	211	0.95 (0.73, 1.23)	0.90 (0.68, 1.18)	0.90 (0.68, 1.18)
50–51	183	1	1	1
52–54	139	0.79 (0.60, 1.03)	0.83 (0.63, 1.08)	0.80 (0.62, 1.05)
≥55	140	0.86 (0.67, 1.11)	0.89 (0.70, 1.14)	0.87 (0.68, 1.12)
*P* for trend		<0.001	<0.01	<0.01
Cardiovascular mortality		HR (95% CI)	HR (95% CI)	HR (95% CI)
<40	22	1.43 (1.12, 2.52)	1.30 (1.07, 2.34)	1.26 (1.03, 2.27)
40–44	45	1.24 (0.79, 1.94)	1.18 (0.73, 1.89)	1.16 (0.72, 1.86)
45–49	56	0.77 (0.51, 1.17)	0.75 (0.48, 1.15)	0.74 (0.47, 1.17)
50–51	71	1	1	1
52–54	48	0.56 (0.35, 0.88)	0.59 (0.37, 0.93)	0.58 (0.37, 0.92)
≥55	39	0.70 (0.44, 1.10)	0.71 (0.45, 1.10)	0.70 (0.44, 1.11)
*P* for trend		<0.01	<0.01	<0.05

Model 1, adjusted for age. Model 2, Model 1 + race, education, hormone therapy, parity status, smoking, BMI, age at menarche. Model 3, Model 2 + total cholesterol, triglycerides, high-density lipoprotein cholesterol, and glycated hemoglobin, hypertension.

#### 3.2.2 Dose-response analyses

**Figure 2:**
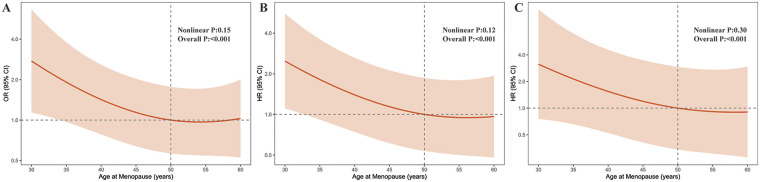
Restricted cubic spline analyses. Associations of age at menopause with cardiovascular disease prevalence **(A)**, all-cause mortality **(B)**, and cardiovascular mortality **(C)** Models were adjusted for age, race, education, hormone therapy, parity status, smoking, BMI, and age at menarche.

Weighted RCS models were used to evaluate the dose–response relationships between age at menopause and cardiovascular disease, all-cause mortality, and cardiovascular mortality. Overall associations were statistically significant (*P* < 0.001), with no evidence of significant nonlinearity for cardiovascular disease (nonlinear *P* = 0.15; Figure 2A), all-cause mortality (nonlinear *P* = 0.12; Figure 2B), or cardiovascular mortality (nonlinear *P* = 0.30; Figure 2C), suggesting an approximately linear relationship.

Weighted logistic regression and Cox regression analyses were conducted. The results showed that each one-year decrease in age at menopause was associated with a 3% increase in cardiovascular disease prevalence (OR = 1.03, 95% CI: 1.01–1.05), a 3% increase in all-cause mortality (HR = 1.03, 95% CI: 1.01–1.04), and a 4% increase in cardiovascular mortality (HR = 1.04, 95% CI: 1.02–1.05). See Table 3.

**Table 3:** Dose–response associations of age at menopause with cardiovascular disease prevalence and mortality.

	Model1	Model2	Model3
Cardiovascular disease prevalence			
OR (per-year decrease)	1.04 (1.02, 1.06)	1.03 (1.01, 1.05)	1.03 (1.01, 1.05)
*P*	<0.001	<0.05	<0.05
All-cause mortality			
HR (per-year decrease)	1.03 (1.02, 1.05)	1.03 (1.01, 1.04)	1.03 (1.01, 1.04)
*P*	<0.001	<0.01	<0.01
Cardiovascular mortality			
HR (per-year decrease)	1.04 (1.02, 1.06)	1.04 (1.02, 1.05)	1.02 (1.01, 1.03)
*P*	<0.001	<0.001	<0.01

Model 1, adjusted for age. Model 2, Model 1 + race, education, hormone therapy, parity status, smoking, BMI, age at menarche. Model 3, Model 2 + total cholesterol, triglycerides, high-density lipoprotein cholesterol, glycated hemoglobin, and hypertension.

### 3.3 Subtype analyses of cardiovascular disease

**Figure 3:**
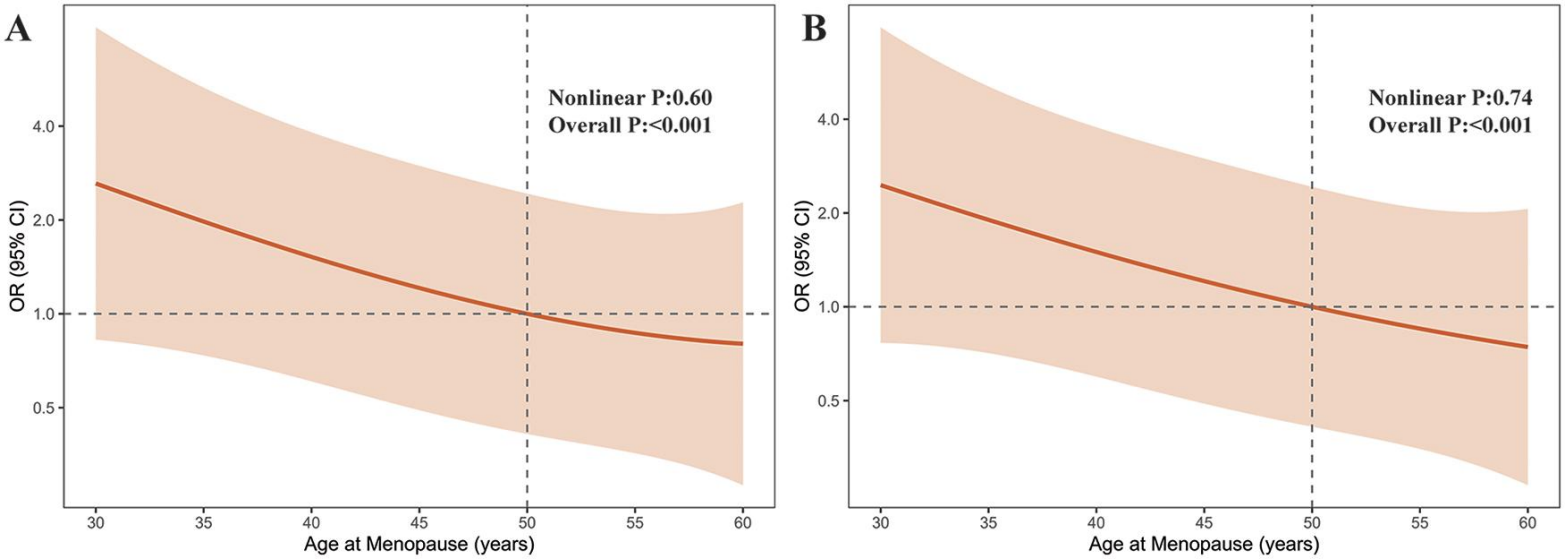
Restricted cubic spline analyses. Associations of age at menopause with coronary heart disease **(A)**, and stroke **(B)** Models were adjusted for age, race, education, hormone therapy, parity status, smoking, BMI, and age at menarche.

Analyses were performed for the major subtypes of cardiovascular disease, including coronary heart disease and stroke. Weighted RCS analyses indicated a linear association between age at menopause and these diseases (Figure 3). Weighted logistic regression analyses showed that each one-year decrease in age at menopause was associated with a 3% increase in coronary heart disease prevalence (OR = 1.03, 95% CI: 1.01–1.05) and a 4% increase in stroke prevalence (OR = 1.04, 95% CI: 1.01–1.07). See Table 4.

**Table 4:** Associations of age at menopause with coronary heart disease and stroke.

	Model1	Model2	Model3
Coronary heart disease			
OR (per-year decrease)	1.04 (1.03, 1.06)	1.03 (1.01, 1.05)	1.03 (1.01, 1.04)
*P*	<0.001	<0.05	<0.05
Stroke			
OR (per-year decrease)	1.05 (1.02, 1.08)	1.04 (1.01, 1.07)	1.04 (1.01, 1.07)
*P*	<0.001	<0.05	<0.05

Model 1, adjusted for age. Model 2, Model 1 + race, education, hormone therapy, parity status, smoking, BMI, age at menarche. Model 3, Model 2 + total cholesterol, triglycerides, high-density lipoprotein cholesterol, glycated hemoglobin, and hypertension.

### 3.4 Subgroup analyses

**Figure 4:**
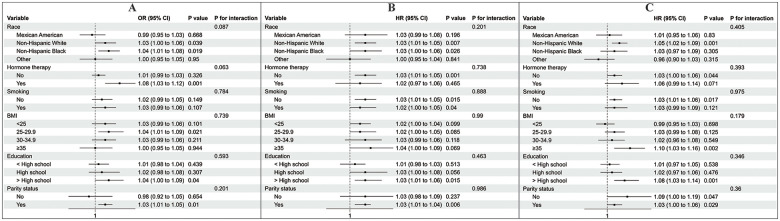
Subgroup analyses. Associations of age at menopause with cardiovascular disease prevalence **(A)**, all-cause mortality **(B)**, and cardiovascular mortality **(C)** estimates represent adjusted ORs or HRs per 1-year decrease in age at menopause across subgroups stratified by race, education, hormone therapy, parity status, smoking, and BMI. Models were adjusted for age, race, education, hormone therapy, parity status, smoking, BMI, and age at menarche, except for the corresponding stratification variable. Dots represent adjusted ORs/HRs, and horizontal lines indicate 95% CIs.

Subgroup analyses were conducted according to race, hormone therapy, smoking, BMI, educational level, and parity status. After adjustment for potential confounders, the associations between age at menopause and cardiovascular disease, all-cause mortality, and cardiovascular mortality were generally consistent across subgroups, suggesting an increased risk associated with early menopause. No significant interactions were observed between subgroups (Figure 4).

### 3.5 Mediation analyses

**Figure 5:**
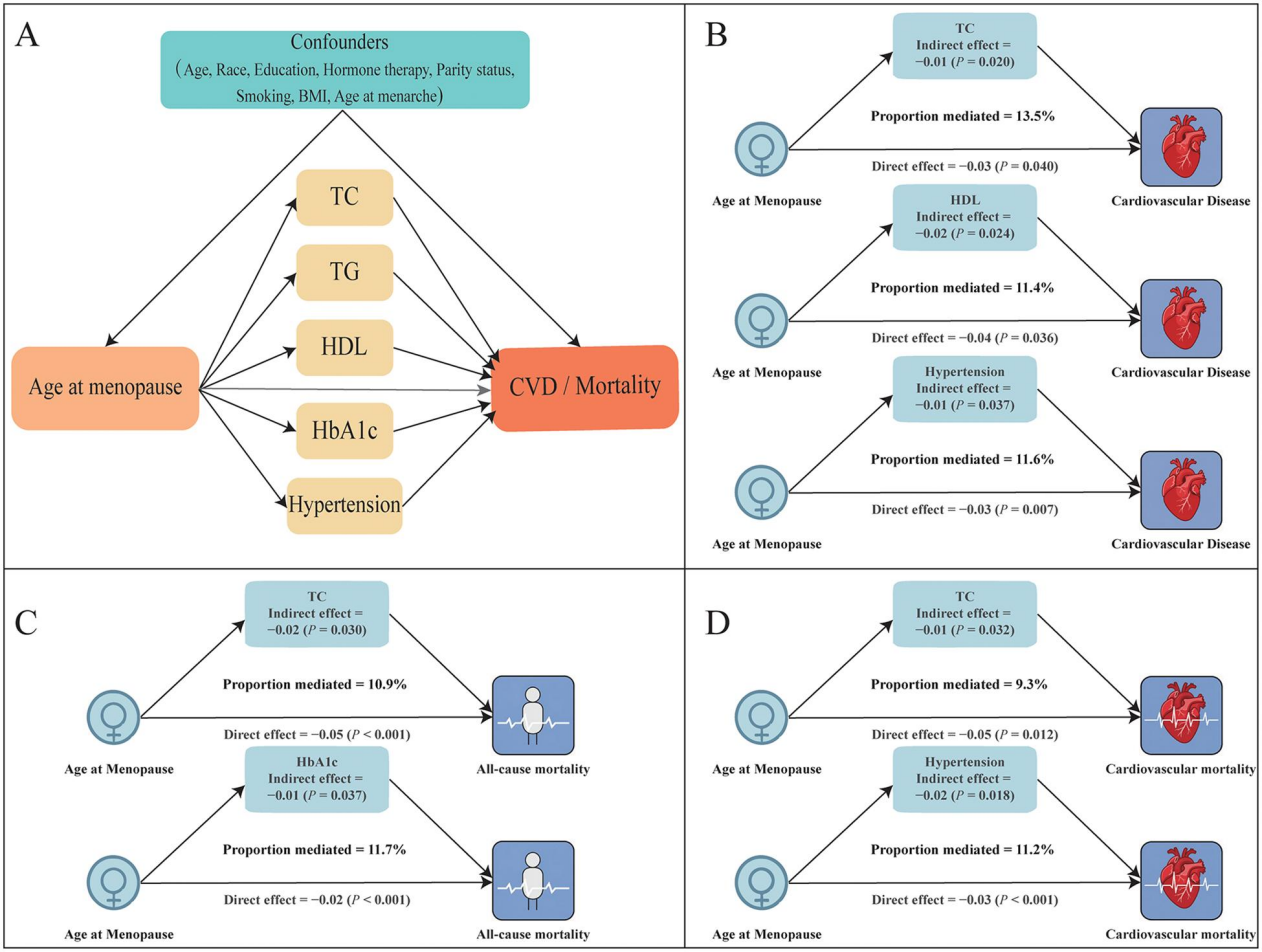
Mediation analyses. Directed acyclic graph **(A)** Mediation analyses of the associations between age at menopause and cardiovascular disease prevalence **(B)**, all-cause mortality **(C)**, and cardiovascular mortality **(D)**.

Mediation analyses indicated that metabolic factors (TC, TG, HDL-C, glycated hemoglobin, and hypertension) played significant mediating roles in the associations between age at menopause and adverse cardiovascular outcomes. TC, HDL-C, and hypertension mediated 13.5%, 11.4%, and 11.6% of the association between age at menopause and cardiovascular disease, respectively. In the analysis of all-cause mortality, the mediation proportions were 10.9% for TC and 11.7% for glycated hemoglobin, respectively. In the analysis of cardiovascular mortality, the mediation proportions were 9.3% for TC and 11.2% for hypertension (Figure 5, Supplementary Table S1).

### 3.6 Sensitivity analyses

Sensitivity analyses showed that, after including both surgical and natural menopause populations, age at menopause was linearly associated with cardiovascular disease, all-cause mortality, and cardiovascular mortality, with each one-year decrease associated with 5%, 4%, and 5% increases in the corresponding risks, respectively. After exclusion of individuals who died within the first year of follow-up, the associations between early menopause and increased risks of the above outcomes remained consistent. Results after exclusion of participants with missing covariates were consistent with those obtained using random forest imputation, further supporting a higher risk of adverse outcomes associated with early menopause. Reclassification analyses showed that, compared with women who experienced menopause at 50–51 years, those with menopause before 45 years had a significantly higher prevalence of cardiovascular disease (OR = 1.42, 95% CI: 1.12–1.88), as well as higher risks of all-cause mortality (HR = 1.31, 95% CI: 1.08–1.71) and cardiovascular mortality (HR = 1.21, 95% CI: 1.05–1.64). See Supplementary Figures S1–S3 and Tables S2–S5.

### 3.7 Supportive analyses in the hospital-based cohort

A total of 1,639 naturally postmenopausal women were included in the hospital-based cohort, with baseline characteristics presented in Table 5. Compared with the NHANES cohort, the demographic and clinical characteristics (e.g., age, age at menarche, and smoking) of the hospital-based cohort were broadly comparable, providing supportive evidence for the primary analysis.

**Table 5:** Baseline characteristics of the hospital-based cohort and NHANES cohort.

Variable	Hospital	NHANES
Age (years)	63.0 (9.3)	64.4 (9.6)
Age at Menarche (years)	12.8 (1.4)	13.0 (1.8)
Age at Menopause (years)	49.4 (5.1)	49.0 (5.3)
BMI (kg/m^2)^		
<25.0	480 (29.3)	1,365 (27.6)
25.0–29.9	512 (31.2)	1,516 (30.7)
30.0–34.9	345 (21.0)	1,116 (22.6)
≥35	302 (18.4)	940 (19.0)
Smoking,n (%)		
No	1,040 (63.5)	3,076 (61.4)
Yes	599 (36.5)	1,932 (38.6)
Parity status,n (%)		
No	150 (9.2)	443 (8.8)
Yes	1,489 (90.8)	4,563 (91.2)
TC (mg/dL)	206.4 (38.1)	207.9 (42.3)
TG (mg/dL)	153.2 (82.9)	149.6 (91.9)
HDL (mg/dL)	58.2 (19.0)	59.6 (17.3)
Hypertension,n (%)	1,010 (61.6)	3,106 (64.8)

BMI, body mass index; TC, total cholesterol; TG, triglycerides; HDL, high-density lipoprotein cholesterol.

**Figure 6:**
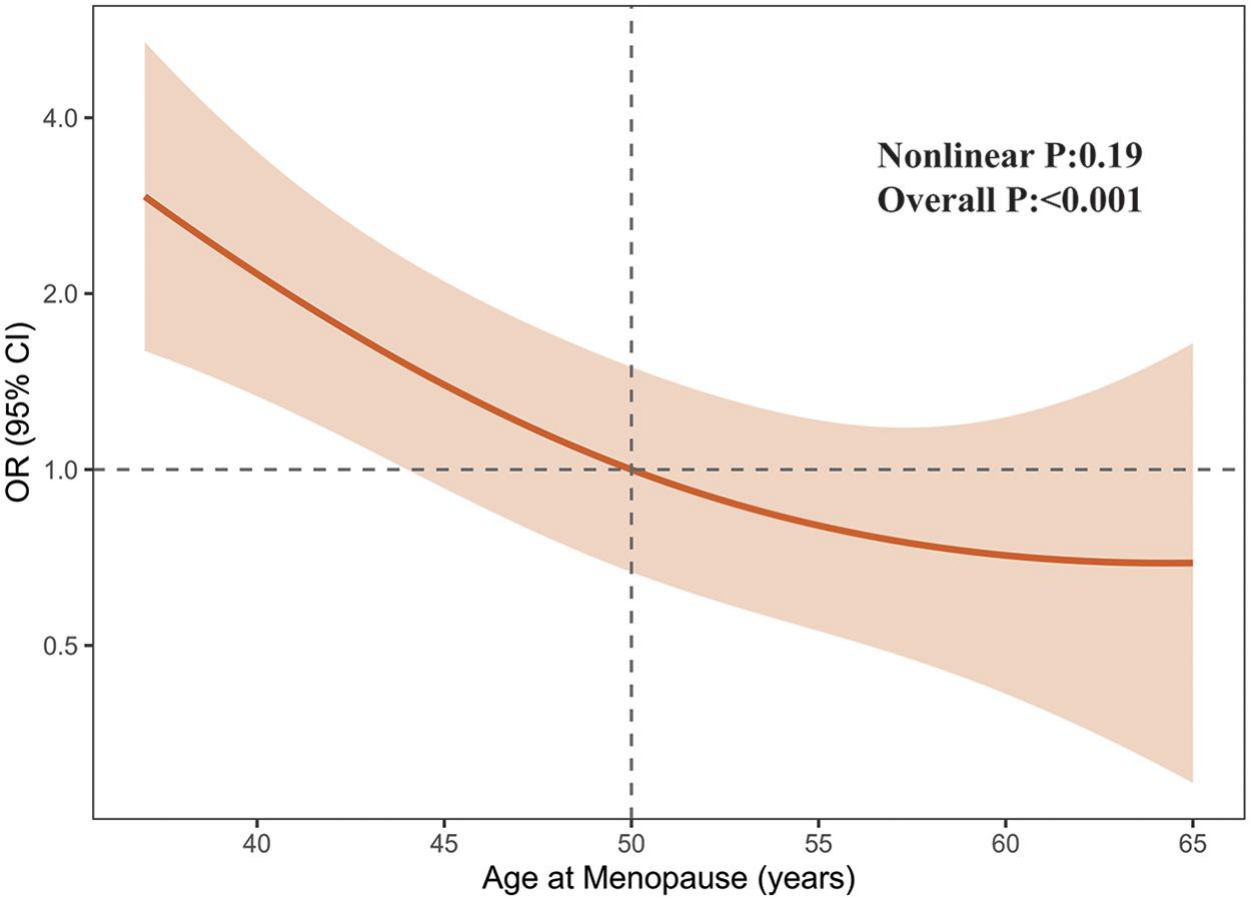
Restricted cubic spline analyses. Associations of age at menopause with cardiovascular disease.

RCS analyses indicated a linear negative association between age at menopause and cardiovascular disease (Figure 6). Logistic regression analysis showed that each one-year decrease in age at menopause was associated with a 5% increase in cardiovascular disease prevalence (OR = 1.05, 95% CI: 1.04–1.07) (Table 6). This association remained consistent after further adjustment for metabolic factors.

**Table 6:** Associations of age at menopause with cardiovascular disease prevalence.

	OR (per-year decrease)	*P*
Model1	1.06 (1.04, 1.08)	<0.001
Model2	1.05 (1.04, 1.07)	<0.001
Model3	1.05 (1.04, 1.06)	<0.001

## 4 Discussion

### 4.1 Association between metabolic dysregulation and cardiovascular disease and mortality

Blood glucose, blood lipids, and blood pressure are important metabolic indicators reflecting systemic metabolic status (12). Studies have shown that hyperglycemia, hyperlipidemia, and hypertension are closely associated with cardiovascular diseases, including coronary heart disease and stroke (13–15). Metabolic dysregulation is associated with increased incidence and mortality of cardiovascular disease (16). Therefore, improving metabolic status may be a potential strategy for reducing the burden of cardiovascular disease. However, the role of metabolic factors in the elevated cardiovascular risk associated with early menopause remains unclear. Therefore, elucidating the role of metabolic factors in postmenopausal women may facilitate the development of targeted prevention and management strategies.

Metabolic dysregulation impairs cardiovascular health through multiple pathological mechanisms. Elevated blood glucose can induce chronic inflammation and oxidative stress, promote atherosclerotic plaque formation, and damage vascular endothelium, thereby increasing the risk of cardiovascular mortality following plaque rupture (17, 18). Different lipid parameters exert distinct effects. Elevated low-density lipoprotein (LDL) promotes foam cell formation and accelerates atherosclerotic progression (19). Reduced HDL weakens its anti-inflammatory and antioxidant effects, thereby aggravating endothelial injury (20). Elevated TG levels may induce acute pancreatitis, thereby increasing the risk of all-cause mortality (21, 22). Hypertension can damage major target organs, including the heart, brain, and kidneys, markedly increase cardiac workload, and ultimately lead to heart failure (23, 24). Collectively, metabolic dysregulation severely impairs the cardiovascular system, highlighting the clinical importance of prevention and management strategies targeting metabolic pathways.

Analysis of the NHANES database showed clear trends toward higher blood glucose, lipid levels, and blood pressure, as well as increased proportions of cardiovascular disease, all-cause mortality, and cardiovascular mortality across earlier age-at-menopause groups, suggesting that metabolic factors may play an important role in early menopause-related cardiovascular disease and serve as potential therapeutic targets.

### 4.2 Association between age at menopause and metabolic dysregulation

Metabolic status deteriorates significantly after menopause, and early menopause may further exacerbate these alterations (25). Postmenopausal women exhibit an increased prevalence of obesity and a shift in fat distribution toward visceral adiposity, thereby contributing to dyslipidemia and insulin resistance (26, 27). The risks of diabetes, hyperlipidemia, and hypertension are significantly increased in postmenopausal women (28, 29). These metabolic disorders are important risk factors for cardiovascular disease (30). Therefore, the increased cardiovascular risk associated with early menopause may be closely linked to metabolic dysregulation.

This study included naturally postmenopausal women for analysis. The results showed that earlier age at menopause was associated with higher proportions of severe obesity (BMI ≥35 kg/m^2^), as well as progressively increased glucose, lipid, and blood pressure levels, suggesting more severe metabolic dysregulation among women with early menopause. Given the important role of metabolic abnormalities in cardiovascular disease and mortality, mediation analyses were performed to explore the potential underlying mechanisms. In addition, TC, TG, HDL-C, glycated hemoglobin, and hypertension were further adjusted for in Model 3 to evaluate whether the association between early menopause and cardiovascular risk changed after controlling for metabolic factors, thereby providing evidence for comprehensive intervention strategies in postmenopausal women.

### 4.3 Association of age at menopause with cardiovascular disease and mortality

Cardiovascular disease included coronary heart disease, congestive heart failure, angina, myocardial infarction, and stroke (31). Coronary heart disease, angina, and myocardial infarction share a common atherosclerotic etiology, whereas stroke is primarily associated with vascular rupture and heart failure results from impaired cardiac pump function (32–34). Although these diseases differ in pathogenesis, their pathophysiological processes are closely interconnected. Atherosclerosis can reduce vascular compliance and increase peripheral resistance, thereby elevating the risk of vascular rupture during blood pressure fluctuations while simultaneously increasing cardiac afterload and exacerbating cardiac dysfunction (35). This study analyzed the associations between age at menopause and cardiovascular disease and its major subtypes, including coronary heart disease and stroke. The results showed a higher prevalence of these diseases among women with early menopause, further strengthening the robustness of the findings. In addition, women with early menopause exhibited significantly increased risks of all-cause and cardiovascular mortality, suggesting that early menopause may adversely affect longevity in women.

Sensitivity analyses were conducted by including both naturally and surgically postmenopausal women, and the results were compared with those from analyses restricted to naturally postmenopausal women. The results showed that, regardless of menopause type, earlier age at menopause was associated with higher cardiovascular disease prevalence, all-cause mortality, and cardiovascular mortality, with stronger associations observed in the overall population analysis. This difference may be related to the younger age at menopause among women with surgical menopause, as well as the potential contribution of underlying diseases requiring surgery to increased cardiovascular risk. These findings suggest that women with surgical menopause may require closer health management and cardiovascular risk monitoring.

A hospital-based cohort was further included for supportive analysis, and the results were generally consistent with the NHANES findings, further supporting an association between early menopause and increased cardiovascular disease prevalence.

### 4.4 Metabolic dysregulation mediates the associations between age at menopause and cardiovascular disease and mortality

Mediation analyses showed that TC, TG, HDL-C, glycated hemoglobin, and hypertension partially mediated the associations between age at menopause and cardiovascular disease and mortality, suggesting a potential role of metabolic dysregulation in early menopause-related cardiovascular disease. However, the mediation proportions were limited, and the associations between age at menopause and the outcomes remained after further adjustment for metabolic factors in Model 3, suggesting that additional mechanisms beyond metabolic dysregulation may also be involved.

Abrupt estrogen decline, enhanced inflammatory responses, increased oxidative stress, and autonomic dysfunction following menopause may represent potential mechanisms underlying the increased risks of cardiovascular disease and mortality associated with early menopause (36–38). However, these mechanisms require further validation in large prospective cohorts. Therefore, glucose-, lipid-, and blood pressure-lowering interventions alone may be insufficient to fully reduce cardiovascular risk in women with early menopause, and comprehensive strategies, including hormone replacement therapy and anti-inflammatory or antioxidant interventions, warrant further investigation.

## 5 Conclusion

Based on analyses of NHANES data and a supportive hospital-based cohort, early menopause was significantly associated with higher cardiovascular disease prevalence, all-cause mortality, and cardiovascular mortality. Mediation analyses further indicated that metabolic dysregulation partially mediated the associations between early menopause and adverse cardiovascular outcomes. These findings suggest the need for comprehensive intervention strategies in postmenopausal women.

## Data Availability

The original contributions presented in the study are included in the article/Supplementary Material, further inquiries can be directed to the corresponding author.
