# Supplementary material for: Association of age at menopause with cardiovascular disease and mortality: insights from NHANES and a clinical cohort
Source: Front Cardiovasc Med. 2026 Jun 23;13:1797019. doi: 10.3389/fcvm.2026.1797019 (PMC13337413; doi:10.3389/fcvm.2026.1797019)
Supplement: Supplementary file 1 [file Datasheet1.pdf]

Supplementary Table S1.

Mediation analyses of metabolic factors in the associations of age at menopause with cardiovascular disease and mortality

Mediating variables	Total Effect		Direct effect		Indirect effects		Proportion of mediators, %
	Coefficient (95% CI)	*P*	Coefficient (95% CI)	*P*	Coefficient (95% CI)	*P*	
Cardiovascular Disease							
TC (mg/dL)	−0.04 (−0.05, −0.02)	<0.001	−0.03 (−0.04, −0.01)	0.040	−0.01 (−0.02, −0.01)	0.020	13.5%
TG (mg/dL)	−0.03 (−0.05, −0.02)	<0.001	−0.03 (−0.05, −0.02)	<0.001	−0.01 (−0.03, 0.02)	0.360	—
HDL (mg/dL)	−0.05 (−0.06, −0.03)	0.020	−0.04 (−0.05, −0.02)	0.036	−0.02 (−0.03, −0.01)	0.024	11.4%
HbA1c (%)	−0.04 (−0.05, −0.02)	0.020	−0.03 (−0.05, −0.02)	0.020	−0.01 (−0.04, 0.03)	0.630	—
Hypertension	−0.04 (−0.06, −0.02)	<0.001	−0.03 (−0.04, −0.02)	0.007	−0.01 (−0.02, −0.01)	0.037	11.6%
All-cause mortality							
TC (mg/dL)	−0.05 (−0.07, −0.02)	<0.001	−0.05 (−0.06, −0.02)	<0.001	−0.02 (−0.03, −0.01)	0.030	10.9%
TG (mg/dL)	−0.03 (−0.04, −0.02)	<0.001	−0.03 (−0.04, −0.01)	<0.001	−0.01 (−0.01, 0.01)	0.684	—
HDL (mg/dL)	−0.04 (−0.05, −0.02)	<0.001	−0.03 (−0.04, −0.01)	<0.001	−0.01 (−0.01, 0.01)	0.468	—
HbA1c (%)	−0.04 (−0.06, −0.01)	<0.001	−0.02 (−0.03, −0.01)	<0.001	−0.01 (−0.02, −0.01)	0.037	11.7%
Hypertension	−0.05 (−0.06, −0.03)	0.007	−0.04 (−0.05, −0.02)	0.012	−0.02 (−0.03, 0.01)	0.161	—
Cardiovascular mortality							
TC (mg/dL)	−0.05 (−0.07, −0.03)	0.012	−0.05 (−0.06, −0.02)	0.012	−0.01 (−0.03, −0.01)	0.032	9.3%
TG (mg/dL)	−0.04 (−0.07, −0.01)	<0.001	−0.03 (−0.07, −0.01)	<0.001	−0.01 (−0.02, 0.01)	0.490	—
HDL (mg/dL)	−0.05 (−0.06, −0.02)	<0.001	−0.04 (−0.06, −0.01)	<0.001	−0.02 (−0.04, 0.02)	0.260	—
HbA1c (%)	−0.05 (−0.06, −0.03)	<0.001	−0.04 (−0.06, −0.02)	<0.001	−0.02 (−0.04, 0.01)	0.240	—
Hypertension	−0.05 (−0.06, −0.02)	<0.001	−0.03 (−0.05, −0.02)	<0.001	−0.02 (−0.03, −0.01)	0.018	11.2%

Analyses were adjusted for age, race, education, hormone therapy, parity status, smoking, BMI, and age at menarche. TC, total cholesterol; TG, triglycerides; HDL, high-density lipoprotein cholesterol; HbA1c, glycated hemoglobin.

Sensitivity analysis 1: Inclusion of women with surgical and natural menopause.

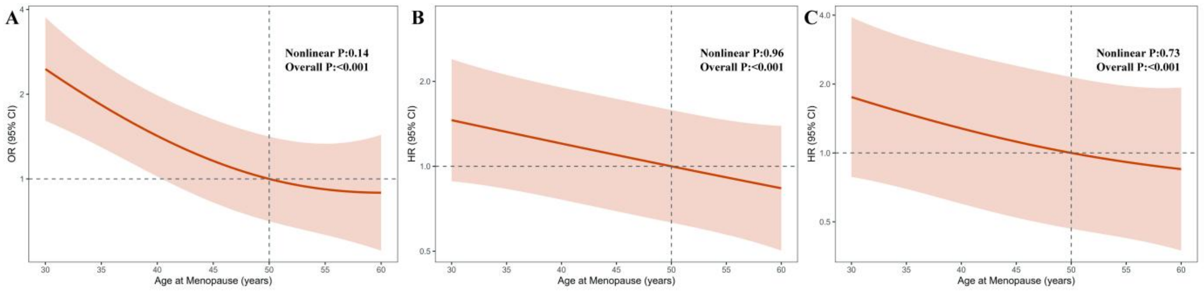

Supplementary Figure S1. Restricted cubic spline analyses. Associations of age at menopause with cardiovascular disease prevalence (A), all-cause mortality (B), and cardiovascular mortality (C). Models were adjusted for age, race, education, hormone therapy, parity status, smoking, BMI, and age at menarche.

Supplementary Table S2.

Dose–Response	N	Model1	Model2	Model3
Cardiovascular disease	1341			
OR (per-year decrease)		1.06 (1.04, 1.07)	1.05 (1.03, 1.06)	1.03 (1.02, 1.04)
P		<0.001	<0.001	<0.001
All-cause mortality	1547			
HR (per-year decrease)		1.05 (1.03, 1.06)	1.04 (1.02, 1.05)	1.02 (1.01, 1.03)
P		<0.001	<0.001	<0.001
Cardiovascular mortality	498			
HR (per-year decrease)		1.05 (1.04, 1.06)	1.05 (1.03, 1.06)	1.02 (1.01, 1.03)
P		<0.01	<0.01	<0.01

Model 1: Adjusted for age. Model 2, Model 1 + race, education, hormone therapy, parity status, smoking, BMI, age at menarche. Model 3, Model 2 + total cholesterol, triglycerides, high-density lipoprotein cholesterol, glycated hemoglobin, and hypertension.

Sensitivity analysis 2: Exclusion of participants who died within the first year of follow-up.

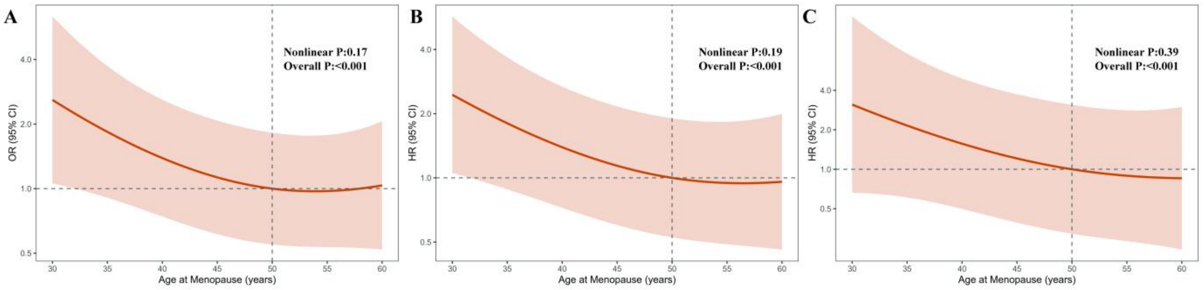

Supplementary Figure S2. Restricted cubic spline analyses. Associations of age at menopause with cardiovascular disease prevalence (A), all-cause mortality (B), and cardiovascular mortality (C). Models were adjusted for age, race, education, hormone therapy, parity status, smoking, BMI, and age at menarche.

Supplementary Table S3.

Dose–Response	N	Model1	Model2	Model3
Cardiovascular disease	634			
OR (per-year decrease)		1.03 (1.01, 1.06)	1.03 (1.01, 1.05)	1.02 (1.01, 1.05)
P		<0.01	<0.05	<0.05
All-cause mortality	781			
HR (per-year decrease)		1.03 (1.02, 1.05)	1.03 (1.01, 1.04)	1.03 (1.01, 1.04)
P		<0.001	<0.01	<0.01
Cardiovascular mortality	253			
HR (per-year decrease)		1.05 (1.02, 1.07)	1.04 (1.01, 1.07)	1.04 (1.01, 1.06)
P		<0.001	<0.01	<0.05

Model 1: Adjusted for age. Model 2, Model 1 + race, education, hormone therapy, parity status, smoking, BMI, age at menarche. Model 3, Model 2 + total cholesterol, triglycerides, high-density lipoprotein cholesterol, glycated hemoglobin, and hypertension.

Sensitivity analysis 3: Exclusion of participants with missing covariates.

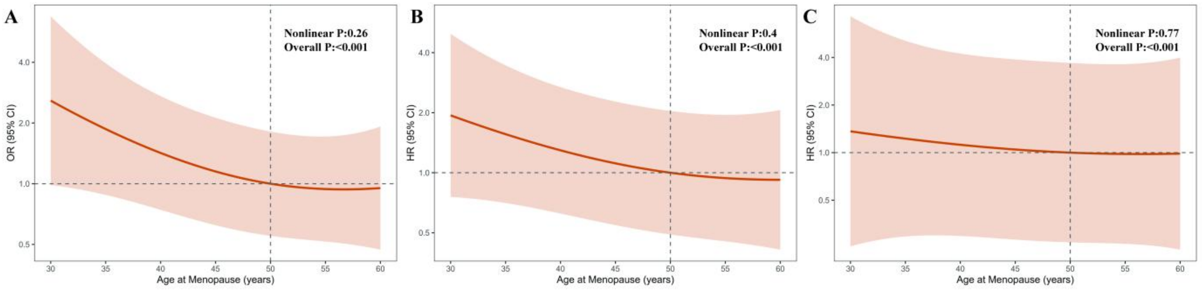

Supplementary Figure S3. Restricted cubic spline analyses. Associations of age at menopause with cardiovascular disease prevalence (A), all-cause mortality (B), and cardiovascular mortality (C). Models were adjusted for age, race, education, hormone therapy, parity status, smoking, BMI, and age at menarche.

Supplementary Table S4.

Dose–Response	N	Model1	Model2	Model3
Cardiovascular disease	569			
OR (per-year decrease)		1.04 (1.02, 1.06)	1.03 (1.01, 1.05)	1.03 (1.01, 1.04)
P		<0.001	<0.05	<0.05
All-cause mortality	678			
HR (per-year decrease)		1.04 (1.01, 1.05)	1.02 (1.01, 1.04)	1.02 (1.01, 1.03)
P		<0.01	<0.05	<0.05
Cardiovascular mortality	222			
HR (per-year decrease)		1.03 (1.02, 1.05)	1.02 (1.02, 1.03)	1.01 (1.01, 1.02)
P		<0.01	<0.05	<0.05

Model 1: Adjusted for age. Model 2, Model 1 + race, education, hormone therapy, parity status, smoking, BMI, age at menarche. Model 3, Model 2 + total cholesterol, triglycerides, high-density lipoprotein cholesterol, glycated hemoglobin, and hypertension.

Sensitivity analysis 4: Reclassification of age at menopause.

Supplementary Table S5.

Cardiovascular disease prevalence and mortality by age at menopause

Age at menopause	Number	Model1	Model2	Model3
Cardiovascular disease prevalence		OR (95%CI)	OR (95%CI)	OR (95%CI)
<45	140	1.50 (1.18, 2.07)	1.42 (1.12, 1.88)	1.34 (1.08, 1.83)
45-49	164	0.90 (0.64, 1.28)	0.85 (0.59, 1.22)	0.83 (0.57, 1.19)
50-51	159	1	1	1
≥52	207	0.80 (0.57, 1.12)	0.82 (0.58, 1.15)	0.81 (0.58, 1.14)
p for trend		<0.001	<0.01	<0.05
All-cause mortality		HR (95% CI)	HR (95% CI)	HR (95% CI)
<45	180	1.38 (1.12, 1.81)	1.31 (1.08, 1.71)	1.29 (1.06, 1.69)
45-49	211	0.95 (0.73, 1.23)	0.90 (0.68, 1.18)	0.89 (0.67, 1.18)
50-51	183	1	1	1
≥52	279	1.11 (0.66, 1.03)	1.11 (0.69, 1.07)	0.84 (0.65, 1.05)
p for trend		<0.01	<0.05	<0.05
Cardiovascular mortality		HR (95% CI)	HR (95% CI)	HR (95% CI)
<45	64	1.28 (1.07, 1.78)	1.21 (1.05, 1.64)	1.19 (1.02, 1.58)
45-49	68	0.77 (0.50, 1.17)	0.74 (0.48, 1.15)	0.75 (0.48, 1.17)
50-51	69	1	1	1
≥52	80	0.63 (0.42, 1.13)	0.65 (0.44, 0.97)	0.65 (0.43, 0.97)
p for trend		<0.01	<0.01	<0.05

Model 1: Adjusted for age. Model 2, Model 1 + race, education, hormone therapy, parity status, smoking, BMI, age at menarche. Model 3, Model 2 + total cholesterol, triglycerides, high-density lipoprotein cholesterol, glycated hemoglobin, and hypertension.
